# Rapid review of decision-making for place of care and death in older people: lessons for COVID-19

**DOI:** 10.1093/ageing/afaa289

**Published:** 2020-12-18

**Authors:** Emily West, Kirsten Moore, Nuriye Kupeli, Elizabeth L Sampson, Pushpa Nair, Narin Aker, Nathan Davies

**Affiliations:** Marie Curie Palliative Care Research Department, Division of Psychiatry, University College London, London, UK; Marie Curie Palliative Care Research Department, Division of Psychiatry, University College London, London, UK; Marie Curie Palliative Care Research Department, Division of Psychiatry, University College London, London, UK; Marie Curie Palliative Care Research Department, Division of Psychiatry, University College London, London, UK; Barnet, Enfield and Haringey Mental Health Liaison Service, North Middlesex University Hospital NHS Trust, London, UK; Centre for ageing population Studies, Research Department of Primary Care and Population Health, University College London, London, UK; Centre for ageing population Studies, Research Department of Primary Care and Population Health, University College London, London, UK; Marie Curie Palliative Care Research Department, Division of Psychiatry, University College London, London, UK; Centre for ageing population Studies, Research Department of Primary Care and Population Health, University College London, London, UK

**Keywords:** palliative care, decision-making, COVID-19, place of care/place of death, advance care planning, older people

## Abstract

**Introduction:**

The coronavirus pandemic (COVID-19) has affected the functioning and capacity of healthcare systems worldwide. COVID-19 has also disproportionately affected older adults. In the context of COVID-19, decision-making surrounding place of care (PoC) and place of death (PoD) in older adults involves significant new challenges.

**Aims:**

To explore key factors that influence PoC and PoD decisions in older adults. A secondary aim was to investigate key factors that influence the process and outcome of these decisions in older adults. To apply findings from current evidence to the context of COVID-19.

**Methods:**

Rapid review of reviews, undertaken using WHO guidance for rapid reviews for the production of actionable evidence. Data extracted was synthesised using narrative synthesis, with thematic analysis and tabulation.

**Results:**

10 papers were included for full data extraction. These papers were published between 2005 and 2020. Papers included discussed actual PoD, as well as preferred. Results were divided into papers that explored the process of decision-making, and those that explored decision-making outcomes.

**Conclusions:**

The process and outcomes of decision-making for older people are affected by many factors—all of which have the potential to influence both patients and caregivers experience of illness and dying. Within the context of COVID-19, such decisions may have to be made rapidly and be reflexive to changing needs of systems and of families and patients.

## Key points

- Preparedness and a sense of control were found to be important for both patients and their families in making decisions.
- Decisions should be considered continually over time, as illnesses progress and priorities and capacities change.
- Appropriate multidisciplinary professional involvement can aid both good decision-making and facilitate patients and families to achieve their stated goals in terms of place of care and place of death.
- Appropriateness of available information in terms of cultural, language and access needs, was shown to be key in empowering family caregivers to cope well with decision-making and caring at home—should this be the preference.
- Sensitivity to cultural appropriateness is especially important in issues surrounding capacity and the role of proxies.

## Background

The coronavirus pandemic (COVID-19) has affected the functioning and delivery of health and social care worldwide. Palliative care (PC) must still be provided amidst the wider surge of demand for services, and rapidly changing needs for triage and service allocation, navigated within the context of rapidly changing situations and guidelines [1].

If an older person becomes unwell, rapid decisions may have to be made concerning place of care (PoC), social distancing, availability of services and which treatments a person may or may not need to receive. Difficult contexts are being navigated such as a lack of visiting opportunities for families in certain care settings [2], decision-making around resuscitation and informational awareness of the current situation. Many older people will not have made an advance care plan or engaged in discussions about their end-of-life care preferences prior to the pandemic [3]. These decisions will often be left for the family to make with practitioners if the older person becomes unwell and/or loses capacity.

Older people may have dementia or other concurrent diagnoses that result in diminished capacity. We know from studies outside the context of a global pandemic that families find making end-of-life decisions for someone who lacks capacity difficult and complex [4,5]. In the UK, almost a third of all COVID-19 deaths were older people living in care homes—many of whom had dementia [6]. Figures from the Office for National Statistics have shown that dementia was the main underlying condition for COVID-19 deaths up to June 2020 [7].

Any decisions made regarding PoC will impact the person who is unwell, their family and caregivers. Families may benefit from support to make decisions using simple decision-making aids. Decision aids provide information about available options, facilitate shared decision-making and have shown to be successful in older populations—including people living with dementia [3,8]. This review is part of a wider study aimed at developing a decision-aid to facilitate making difficult decisions with people living with dementia, and their family and caregivers in the context of the COVID-19 pandemic. Active participation in decision-making at the end of life can positively impact on caregivers’ experience of grief, through increased feelings of deliberateness and inclusivity in the dying process [9].

The primary research question of this rapid review-of-reviews is ‘which factors influence place of care and place of death decisions in older adults?’ A secondary research question is ‘which factors influence the process and outcome of these decisions in older adults?’ The findings from this review of current evidence will then be explored through the context of healthcare challenges during COVID-19.

## Methods

**Figure 1:**
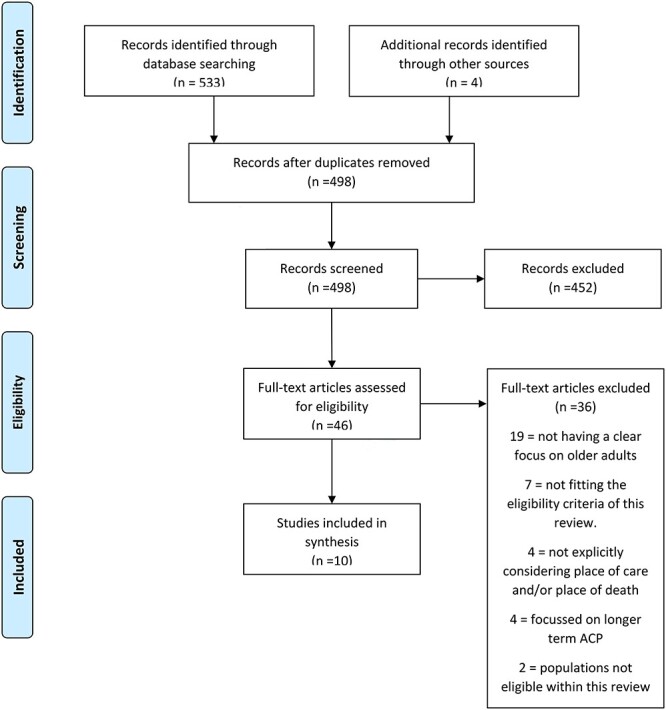
PRISMA diagram.

This review is situated within the context of COVID19, which is a rapidly developing situation and in circumstances such as these the WHO recommends the use of rapid reviews for the production of actionable evidence [10]. This rapid review of reviews was undertaken according to 2017 WHO guidance [10] and reported according to the Preferred Reporting Items for Systematic Reviews and Meta-Analyses guidelines; see Figure 1.

### Inclusion criteria

Papers included were systematic and narrative reviews, including meta-analyses. Papers focussed on older people—patients over the age of 65, professionals, caregivers and the general population who were concerned with caring for people over the age of 65. There were no language limits specified, though the search was conducted in English.

### Search strategy

We searched two databases (MEDLINE [1966–2020] and Embase [1980–2020]) as well as grey literature and clinical guidelines on 22nd April 2020. The search strategy comprised terms for end-of-life and PC, as well as older adults, care settings and decision-making. We identified and screened the reference lists of relevant identified reviews and consulted experts in this field.

### Study selection

One researcher (EW) completed searches and de-duplicated results. Titles and abstracts were screened by EW and NA, and 50% were checked by the wider team. Full-texts were screened by EW and NA and all included papers were ratified by the wider team. Data extraction was performed by EW. Due to the rapid nature, quality was not appraised. Concerning risk-of-bias, WHO guidelines note that ‘when the purpose of a review is to scope the available literature, rather than to evaluate specific effects, this step may not be needed’ and thus risk-of-bias has not been included formally, though concerns around this topic are later discussed.

### Data extraction

Articles were managed in Mendeley. Study aims, design, population and setting, objectives and main findings were included in the primary data extraction table.

### Synthesis

Data extracted was analysed through narrative synthesis and themed based on several major categories—namely care settings, disease groups, study population, decision-making approach (formal approaches such as advanced care planning, as well as informal modes such as conversations) and decision-making role (whether informational, supportive etc.). These themes were devised iteratively throughout the review process by EW and ratified by the wider team.

## Results

We identified 533 papers from database searches up to 22 April 2020 and eliminated 39 duplicates. Four further papers were found through reference searches and 46 papers were included for full-text review. Nineteen were excluded for not having a clear focus on older adults, four for not explicitly considering PoC and/or place of death (PoD) and seven for not fitting the eligibility criteria of this review.

### Description of included papers

Articles included for analysis approached the research question from a number of different standpoints. The evidence primarily spanned North America and Europe, though as included papers were themselves systematic reviews, there was considerable variation within papers.

The 10 papers included for data extraction were published between 2005 and 2020. Table 1 shows an overview of the range of themes.

**Table 1:** Data extraction table of the main points of included studies

	Howelll	Hoare	Gomes	Costa	Tejedor	Hudson	De Souza	Kwak	Goodman	McCaffrey
Preference PoD/PoC	x	x	x	x	x	x	x	x	x	x
Actual PoC/PoD		x	x	x	x	x			x	
Home care/death	x	x	x	x	x				x	x
Hospital care/death	x	x		x	x	x			x	x
Hospice care/death		x								x
Nursing home care/death		x		x				x	x	
Cancer	x	x	x	x		x		x		x
Non-cancer		x	x	x		x		x		x
Dementia								x	x	
Patient views		x	x	x	x	x	x	x	x	
Caregiver views		x	x	x	x	x	x	x	x	
General Population		x	x							
BME focus							x	x		
Conversations					x		x	x		
Decision-making	x	x	x		x		x	x	x	
ACP/ad				x	x		x	x	x	
Specialist PC	x			x	x				x	x
Service design/access	x			x		x			x	x
Information						x				
Support						x				
Capacity					x				x	

Abbreviations: BME, Black and Minority Ethnic.

Six papers discussed actual PoD, as well as preferred [11–16]. Seven papers explored home as a PoD [11–14,16–18]. Seven papers looked at hospital deaths [11,13–18]. Two papers looked at hospice as a setting [11,18] and four at nursing homes [11,13,16,19]. There were differences in diagnoses included with seven papers looking specifically at patients with cancer [11–13,15,17–19], six including non-cancer diagnoses [11–13,15,18,19] and two looking specifically at dementia [16,19].

Eight papers explored the subject through patient and caregiver views [11–16,19,20], two included general population views [11,12] and two had a specific minority ethnic focus [19,20].

Three papers looked explicitly at conversations surrounding decision-making at the end of life [14,19,20]. Seven papers considered the decision-making process through using future or hypothetical scenarios to explore the decision-making process [11,12,14,16,17,19,20] and five discussed elements of advance care planning (ACP) and advance decisions [13,14,16,19,20].

Five papers examined the role of specialist PC [13,16–18]. Five looked explicitly at service design and access to care [13,15–18]. One paper [15] looked at the role of information and support in decision-making, and two [14,16] explored issues surrounding capacity.

In performing data extraction, a split in the focus of papers emerged between those that primarily concerned on the process of decision-making (Table 2) and those that concerned the outcomes of decision-making (Table 3). This two-theme approach was devised iteratively, as data extracted showed a clear split in focus between those that looked at decision-making as something that had already taken place, and those where decision-making was a process in progress or a hypothetical future scenario. To give a clear overview of these different approaches, we split the tables according to those two.

**Table 2:** Process elements of decision-making

Author, year, country, journal	Study design	Population and setting	Objectives	Main findings
Hudson, Best, Stone, Noble 2019	Rapid review Original peer reviewed studies published in English between June 2008 and June 2018. Primary qualitative, quantitative or mixed methods interventions exploring the impact of continuity in palliative care were eligible for inclusion.	1951 patients and 190 family caregivers were recruited across included studies. Interventions recruiting adults (aged over 18 years) receiving palliative care and/or their family, friends or carers. Participants at all stages of a terminal illness, including the dying phase were included. Most studies (*n* = 10) recruited patients with a range of illnesses, identified as requiring palliative care, three studies recruited patients with cancer, while three recruited patients with a different diagnosis (chronic obstructive pulmonary disease, Parkinson’s disease and advanced heart failure). Two studies recruited bereaved family members. Half of included studies were conducted in the UK (50%, *n* = 9), three were conducted in the USA or Canada, two in Australia and one each in Iceland, the Netherlands, Sweden and Denmark.	To identify the potential impact of continuity on their experiences of care. To explore the impact of interventions designed to promote continuity for people receiving palliative care on achieving preferred PoD, reducing avoidable hospital admissions and satisfaction with care.	Over half of interventions identified explored impact on PoD. Two interventions reported a positive impact on facilitating preferred PoD, while this was difficult to assess in two interventions due to a lack of comparator or limited information being reported. No studies described a negative impact, or a decrease in the number of deaths occurring in the preferred locations. All interventions included promoted relational continuity, and the majority included liaison between medical teams (informational continuity). Six studies explored the impact of intervention on hospital admission rates. Most described a reduction in avoidable hospital admissions for people enrolled in interventions. Participants described a significant impact on their experiences as a result of the lack of informational and relational continuity. Patients and carers described difficulty in navigating the numerous services and multiple people involved in their care. Many described uncertainty about how and when to access support. Delays in seeking support out of hours were common, due to lack of informational continuity and uncertainty surrounding their use.
De Souza, Gillett, Froggatt, Walshe 2020	Systematic literature review and meta-ethnography. Studies of conversations between ethnic minority older people and family about end-of-life care published between 2005 and 2019.	13 studies from the UK, USA and Canada.	To develop a deeper understanding of the perspectives of older people of BME heritage and their children, about end-of-life conversations that take place within the family, using a meta-ethnographic approach. To explore how these experiences have on the way these older people feel and think about initiating discussions about their end-of-life preferences?	Four main themes concerning EoL conversations were found: ‘My family will carry out everything for me; it is trust’, ‘No Mum, don’t talk like that’, ‘I leave it in God’s hands’ and ‘Who’s going to look after us?’ All studies shared a common theme of the pivotal role of family in end-of-life decisions. The belief that—‘*my family will do right by me*’—conveys trust in their children’s role as carers and decision makers for them at the end of life. Some older people were concerned that they were creating suffering and burden merely by discussing end-of-life issues. The study found that people from BME heritage appear to respond poorly to generic end-of-life planning initiatives, though those who had been exposed to specifically targeted culturally adapted education demonstrated a desire for engaging with more formal forms of advanced care planning.
Kwak, Haley 2005	Narrative literature review American and Canadian studies from between 1992 and 2003.	33 studies from Canada and the USA. The participants of these studies were recruited from community and senior centers, managed care settings, nursing homes, Alzheimer’s disease clinics, and outpatient and inpatient medical settings, hospitalised medicare beneficiaries, community-dwelling older adults, and family members of deceased older people. The studied racial or ethnic groups included older adults designated as African American, Asian, American Indians, Chinese, Filipino, Hawaiian, Hispanic, Iranian, Japanese, Jewish, Korean and Mexican American.	To explore variations within groups related to cultural values, demographic characteristics, level of acculturation and knowledge of end-of-life treatment options.	12 studies examined the AD among different racial or ethnic groups. Two studies found no difference between three racial or ethnic groups including African American patients, whereas 9 other studies found that African Americans were less likely to complete AD. This finding was consistent across community-dwelling older adults, nursing home residents, cancer patients and physicians. African American, Hispanic and White adult patients did not differ in their desire to discuss end-of-life-care options with physicians, and the majority of participants from all three groups wanted this to take place during routine clinic visits Two studies examined informal end-of-life-care communication among older adults and their family members. There was no difference between Whites and African Americans in these preferences. African American decedents were less likely than White decedents to have had an informal communication about preferences regarding health care with their family members.
				Mexican American older adults were more often found to prefer having family members making decisions regarding end-of-life treatments compared with White or African Americans Hispanic older adults were less likely than Whites or African Americans to believe that a formally designated health care proxy was needed when family was involved in the medical setting. African American and Hispanic patients were most likely to have a daughter as an alternative decision maker, Asians were more likely to have a son and White patients were most likely to have a spouse as an alternative decision-maker.
Goodman, Evans, Wilcock, Froggatt, Drennan, Sampson, Blanchard, Bissett, Iliffe 2010	Integrated review Published and unpublished English language studies on palliative care for older people with dementia, from between 1985 and 2006 Grey literature and hand searches of non-indexed and frequently cited journals were included.	67 research papers. 61% from North America, 38% from Europe (including the UK) and the remainder from Asia and Australia. 64% were from the care home setting. 18% exclusively concerned living and dying with dementia at home. 9% included at home care in mixed settings.	To review the evidence for end-of-life care for community dwelling older people with dementia (including those resident in care homes).	Four main themes were identified: the experience of caring for dying people with dementia, how dying in people with dementia is recognised, symptom management and decision-making about end-of-life care for people with dementia. The highest proportion of papers (41%) focused on describing dementia care towards the end of life. Predicting the approach of death for people with dementia (18%) was a particular focus of the USA owing to rules surrounding financial eligibility for different kinds of care. 25% of studies looked at practitioners’ and family carers’ EoL decision-making and a further 25% at advanced care directives. 19% explored pain and behavioural symptoms, discomfort and the effects of with-holding nutrition and hydration. Studies that compared end-of-life care for people with dementia between countries and settings found significant differences between symptoms, physician responses and patient outcomes. People with dementia living in nursing homes were found to experience fewer adverse symptoms and improved levels of comfort when the care was dementia specific. People with dementia were found to receive less pain relief medical services than those with other diagnoses. Carers’ experiences and responses are heavily affected by dementia-related behaviours and a sense of prolonged loss, Carers are negatively affected by the loss of choice and control when someone moves into a care home or is transferred to hospital One study found that most residents could state a simple treatment preference (82.4%), many did not retain capacity to understand treatment alternatives or grasp the consequences of their choice. Having an advance care plan may affect decision making about potentially life-prolonging interventions. Contextual factors such as level of education, perception of patient QoL, rural or urban settings and nationality all influence individuals’ responses concerning PoC and interventions at the end of life Decision-making for people with dementia at the end of life is also shaped by differences in religious beliefs, professional training, understanding of the disease, what is meant by palliative care, perspectives of other patients, culture and beliefs The majority of older people with dementia live and die at home or in a care home.
McCaffrey, Bradley, Ratcliffe, Currow 2016	Systematic review and synthesis of qualitative research. Published, peer-reviewed, English-language articles reporting primary qualitative data investigating QOL domains in adults with a progressive, life-limiting illness from between database inception and 31st December 2015.	24 studies from between 1992 and 2015. Studies were from 10 countries, most commonly the USA (*n* = 6), England (*n* = 5), Canada (*n* = 3), and Sweden (*n* = 3). Studies included participants diagnosed with cancer (*n* = 16), heart failure (*n* = 2), AIDS (*n* = 2), chronic obstructive pulmonary disease (*n* = 1), or mixed populations (*n* = 3). Most focused on investigating QOL domains (*n* = 17). Seven studies explored the constituents of a good death. Half of the studies recruited from a community setting. Three studies were conducted in a hospice and six studies recruited participants from a mix of settings. 483 individuals from across the studies contributed to the qualitative data analysis.	To identify which aspects of QOL are important from palliative care patients’ perspectives.	Eight important aspects of QOL were identified following framework synthesis: cognitive; emotional; health care; personal autonomy; physical; preparatory; social and spiritual. Two studies included all eight aspects. 23 studies reported that spiritual, physical and social aspects were important. 6 studies highlighted cognitive aspects. Most studies reported emotional aspects (*n* = 20) and aspects relating to personal autonomy (*n* = 19). 13 studies identified the aspects of health care provision, including access, continuity, quality and PoC. 18 studies reported on preparation. The location of health care service provision was noted to be important in QoL, whether. Participants both appreciated the professionalism of hospital care and the familiarity and comfort of home-based care and the chance to die at home. The ability to make choices concerning treatment decisions and daily activities on a palliative care unit gave participants a feeling of control and empowerment, and was important in terms of personal autonomy. Maintaining independence contributed toward a sense of normalcy, whereas diminishing independence led to loss of dignity and feelings of frustration. The pattern of themes did not appear to differ by diagnosis, living arrangements or recruitment setting.

**Table 3:** Outcomes of decision-making

Author, year, country, journal	Study design	Population and setting	Objectives	Main findings
Howell, Roman, Cox, Smith, Patmore, Garry, Howard 2010	Systematic literature review and meta-analysis. Studies included were published between 1966 and 2010.	Adult haematology patients. PoD was assessed using morbidity and mortality data.	To systematically examine all studies of PoD in haematology patients and meta-analyse risk estimates. To look at factors that contribute to hospital death in this population.	Home is found to be the preferred PoD. Haematology patients usually die in hospital. Thus patients may not be dying where they wish. Health commissioners may be funding costly end-of-life care in inappropriate acute hospital settings. More research is needed about preferred PoC for haematology patients, reasons for hospital deaths, and how these can be avoided.
Hoare, Morris, Kelly, Kuhn, Barclay 2015	Systematic literature review. Studies included were published between 2000 and 2015.	21 reports included a range of cancer and non-cancer illnesses. 12 did not state the participants’ illnesses. Of 10 studies of the general population, none had a specific disease focus. 48 reports were of patient preferences and 11 were proxy reports from family carers. One was a study of healthcare professionals. 10 were the surveys of the general population. Data collection was undertaken in hospital (*n* = 10), hospices (*n* = 8), participants’ homes (*n* = 5), care homes (*n* = 2) and in the ‘community’ (*n* = 6). 17 studies were also undertaken in ‘multiple’ settings where participants were asked in either primary and secondary care or where the participant was responding on behalf of a patient, and 10 among the general population where location was not relevant.	To explore preferences for PoD. To compare PoD preferences between the general public and dying patients.	Patients, proxies and public all expressed a majority preference for home when missing data were excluded. When missing data were included, it was not known what proportion of patients or family proxies preferred home. Where patients wished to die was related to where they were asked their preference, and reflected where patients were cared for. Participant preferences for home were more heterogeneous than those expressed by the general public. Neither the general public nor family caregivers appeared to be accurate proxies for patients’ preferences for PoD.
Gomes, Calanzani, Gysels, Hall, Higginson 2013	Systematic literature review. Studies included were published between 1966 and 2011	210 studies reported preferences of over 100,000 people from 33 countries. Included were 34,021 patients, 19,514 caregivers and 29,926 members of the general public.	To examine the heterogeneity in estimates of home-death preference To explore reasons for variation: in relation to the quality of studies, the way in which preferences were assessed and changes of preference with illness progression.	We found moderate evidence that the majority of people prefer dying at home (75% of 130 studies). Based on 10 studies, around four-fifths of patients did not change preference as their illness progressed. Heterogeneity was lowest among general public studies and greatest among patient studies. Preference for home may be less frequent amongst lay caregivers and older people. Home-death preference estimates ranged from 31 to 87% for patients (9 studies), 25 to 64% for caregivers (5 studies), 49 to 70% for the public (4 studies). 20% of 1,395 patients in 10 studies changed their preference, but statistical significance was untested. Qualitative research also revealed a conceptual distinction between preferring home as the PoC and as the PoD.
Costa 2014	Systematic review and meta-analysis. Studies included were published between 2004 and 2013.	Most patients were older than 65 years of age and between 27 and 100% were male. The rate of nursing home death ranged from 47 to 87% in the studies restricted to nursing home residents and from 13 to 26% in the studies of general end-of-life population. The sample sizes ranged from 86 to 181,238 patients.	To evaluate the determinants of PoD for adult patients who have been diagnosed with an advanced, life-limiting condition and are not expected to stabilise or improve.	Factors that increased the likelihood of home death included multidisciplinary home palliative care, patient preference, having an informal caregiver and the caregiver’s ability to cope. Factors increasing the likelihood of a nursing home death included the availability of palliative care in the nursing home and the existence of AD. A cancer diagnosis and the involvement of home care services increased the likelihood of dying in an inpatient palliative care unit.
				A cancer diagnosis and a longer time between referral to palliative care and death increased the likelihood of inpatient hospice death. Determinants that increased the likelihood of a death at home included: interprofessional home end-of-life/palliative care an earlier referral to end-of-life/palliative care services, type of underlying disease, worse functional status, fewer hospitalisations during the last year of life, living arrangements such as living with someone, presence of an informal caregiver, informal caregiver coping and patient or family preference for a home death Determinants that affected a patient’s likelihood of dying in a nursing home included the type of disease, a worse functional status, the availability of palliative/end-of-life services in the nursing home, having completed an advance directive, a longer duration of stay in the nursing home, nursing home bed availability and whether the patient preferred to die there.
Tejedor 2017, Spain	Literature review	Population: Included studies were those focused on very old people or that included people over 85 years old. Setting: Not stated.	The objective of this literature review is to understand very old people’s health care preferences. By this review, its author aims to help improving health outcomes and planning in the healthcare system through very old people’s active engagement.	There are several barriers to the exploration of very old people’s preferences, both from the patient (e.g. patient’s family rejection, passive expectation that others will decide for them and uncertainty about the future) and the professional side (e.g. lack of awareness about ACP, time, communication skills, cultural/emotional/ideological settings and fear to create an ‘anticipatory grief’). Even where there is some communication patient-professional, this communication is not necessary effective as some older people lack the basic abilities to/knowledge to weight pros and cons of treatment. Older people might feel important to talk about their opinions regarding the end of life; however, they do not regularly have these conversations with families. Health values that shape very old people’s preferences—Pain and symptom control (physical wellbeing)—Minimal physical and mental dependency—Not feeling to be a burden—Trust and good communication with the doctor—Be surrounded by family and friends—Personal and business conflicts sorted—Be treated as a ‘whole person’, completing the life circle -Feeling in control over decision-making—Decisions aligned with personal and family preferences—Clear knowledge of an approaching death—Be able to prepare for death Health preferences—Treatment intensity Most studies show that older people would choose quality of life over quantity; and a natural/peaceful death rather than surrounded by life supporting means. Most would chose comfort measures and die at home, however, when they are presented with specific clinical scenarios, older people are more prone to choose life supporting means, which might highlight the lack of knowledge regarding medical procedures that could interfere when picking their preferences. Invasive treatments are preferred when these would lead to significant improvements. Conversations with patients should be hold periodically to reflect their changes of opinion over time. PoD Most would choose to die at home, but this opinion tends to change once the end of life is approaching. Place of treatment Initially they prefer to be treated at home, but again when the end of life is approaching they tend to prefer a specialised setting. Involvement in own care planning Some older people do not want to get so much involved in their decision-making rather than developing a relationship of trust with the doctor. Others however, want to have the necessary information and discuss with the doctors, and take a more active role in their decision-making processes. Improvement suggestions—ACP—Healthcare systems adapted to very old people

### Decision-making process

Five papers focussed on the process aspect of decision-making (Table 2)—papers included in this table focus on the aspects of decision-making, such as service design, access to care, advance directives (AD) and the process of making decisions.

#### Factors affecting decisions

Hudson [15] explored the role of informational continuity on achieving patients’ preferred PoD. This was defined as ‘coordinated, comprehensive information sharing’. No interventions assessed were negatively associated with achieving preferred PoD, but out of 10 papers analysed, only two showed a clear positive affect. Goodman *et al.* [16] looked at patients dying with dementia, the decisions made around their end-of-life care plans and factors affecting this. They found the level of education, perceived quality of life, demographics and understanding of the disease and the role of PC affected decision-making. Most patients in this study lived and died at home, or in a care home. McCaffrey [18] focussed on the role of quality of life in decision-making and recognised that PC settings allowed patients and caregivers to feel a sense of control and autonomy around making care decisions.

#### Emotional aspects of decision-making

Hudson [15] described the emotional impact of poor informational continuity on people with PC needs and their families, highlighting how a lack of continuity and reliable access to good information worsened feelings of vulnerability, and contributed to feelings of being lonely and unsupported. De Souza [20] found a number of incidences where families found discussing future options for PoC and PoD difficult, due to perceived creation of suffering and emotional burden on family members. Goodman *et al.* [16] address emotional involvement in decision-making through exploring loss, particularly in regards to family caregivers. They found that caregivers’ decisions are often heavily shaped by the idea of loss, be that loss of control and identity if a patient moves to a care home or hospital or the way that prolonged loss throughout the course of a long-term illness may shape decisions made. McCaffrey [18] considered feelings of control and empowerment to be particularly important in the case of patients making their own end-of-life care and PoD decisions. Contrary to this, experiencing a lack of control in making such decisions was found to lead to frustration and loss of dignity.

#### Capacity in decision-making

Several papers explicitly addressed the subject of capacity, most often in the sense of capacity being diminished. Though considering capacity in a more abstract sense, De Souza [20] identified the theme ‘my family will do right by me’ as key in examining the preferences of minority ethnic patients and their families in PoC and PoD decision-making. This implicitly suggests a sense of trust that continues despite potential future fluctuations in capacity. Goodman *et al.* [16] specifically focussed on the needs of those dying with dementia and their families, and thus addressed the issue of capacity explicitly throughout. This study found that over 80% of patients could not retain capacity to understand alternatives to expressed treatment preferences, and did not grasp the consequences of their decisions. In discussing issues surrounding capacity, the role of proxy decision-makers was paramount. Kwak [19] found differences between ethnic groups in the family position of nominated proxies. Caucasian patients were most likely to appoint a spouse, Asian-Americans a son and Black and Hispanic patients a daughter. Hispanic patients were less likely to designate a formal proxy decision-maker in their care.

#### Minority experience in decision-making

Two papers specifically assessed the experience and process of end-of-life decision-making for older minority ethnic adults. De Souza [20] looked at the characteristics of decision-making conversations between minority ethnic older people and their families. All of the studies they included highlighted the role of family in decision-making. Importantly, this study found a poor response among minority ethnic older people and their families to generic end-of-life planning initiatives, but a positive response to culturally tailored interventions through natively embedded religious or cultural institutions. Kwak [19] explored the role of AD in decision-making in ethnically or racially diverse groups. African-Americans were found to be less likely to complete AD, regardless of their PoC. There were no differences found between ethnic or racial groups in terms of their desire to discuss end-of-life options with healthcare professionals.

### Decision-making outcomes

Some papers explored the subject of decision-making in terms of outcomes—where people expressed preferences to be cared for and to die, and factors influencing these decisions (Table 3). Often, the line between PoD and PoC was not clear, with respondents and researchers using the terms interchangeably.

#### Place of death

A strong preference for home death was found across the board [11–14,17]. Hoare [11] found that preferences for PoD reflected where the patient was asked the question, and where they had been cared for. Herrera-Tejedor [14] found that older adults generally prioritised a peaceful death with good pain and symptom control, and quality over quantity of life in decisions around dying at home, though when given specific clinical scenarios they were more likely to choose life supporting measures. Howell [17] found a large discrepancy between preferred and actual PoD in haematological malignancy, with the majority dying in hospital despite expressing a preference to die at home. Hoare [11] found that home death was preferred by public, patients and proxies—though patient preferences were more heterogeneous than those of the general public, and neither public nor caregivers appeared to be accurate proxies for patient end-of-life decision-making. Gomes [21] also found a greater degree of heterogeneity between preferences expressed by patients than the public. Preference for death at home was less frequent among older people and caregivers than among professionals and the general population.

#### Decisions over time

Both Gomes [21] and Herrera-Tejedor [14] explored changes in preferences over time, with Gomes finding that the majority of patient preferences did not change over time and Herrera-Tejedor conversely finding that patients preferred home death initially, but this changed as the end-of-life approached to a preference for specialised settings.

#### What affects decisions made?

Costa [13] found that actual incidences of dying at home were positively associated with the involvement of a multidisciplinary home PC team, the patient expressing a clear preference, having an informal caregiver and the caregiver’s ability to cope.

## Discussion

This rapid review was undertaken to explore the range of factors affecting decision-making concerning PoC and PoD among older adults. In the current context of the global COVID-19 pandemic, such decisions are both more acute—as the disease more severely affects those who are older or have multiple comorbidities—and more heightened in the sense that health care systems are working with ever-shifting allocation and triage needs. Literature included in this review explored a diverse breadth of issues, both in terms of outcomes and the process of making such decisions. Decision-making for older adults at the end of life is a complex process which can be affected by myriad factors and, sometimes, competing or conflicting priorities.

### Methodological limitations

Rapid reviews are a somewhat unusual method of research synthesis, often precipitated by urgent health system needs. In line with WHO guidance [10], this review did not include risk-of-bias assessment, as the purpose of the review was to scope available literature rather than to evaluate specific effects. Individual included reviews may show bias in terms of included populations or methodologies, which could influence a meta-review such as this. In light of our use of rapid review methodology, included studies are largely considered as equally robust. Similarly, there are studies, included in this review, that have been picked up by included reviews separately. As this review does not assess results in a cumulative fashion, potential double-counting of included studies is unlikely to have an effect on the overall conclusions of the review. However, more policy or intervention-focussed reviews would need to be wary of this effect. Thus, this review is limited in the degree to which it can be applied to policy decisions.

### PoC and PoD

PoC and PoD, both actual and preferred, proved to be a key theme in decision-making among older adults. Home death was strongly favoured across papers, though this was contingent on factors such as having a caregiver and that caregiver’s ability to cope [13]. Achievement of home death when this is the preferred PoD may necessitate the involvement of specialist, multidisciplinary care teams [13,22]. There is evidence [23] that death at home may necessitate additional support for family caregivers, as they navigate changing concepts of home and ambiguity in bereavement. In the context of COVID-19, caregivers may also be balancing multigenerational caregiving responsibilities, economic insecurity and a lack of usual support networks. This has immediate effects on caregiver well-being, and potentially ability to cope. However, strict and rapidly changing guidelines around local lockdowns and visiting guidelines may also put pressure on decisions concerning PoD and PoC.

### The role of family and culture

The role of family or other proxy decision-makers is a key when considering decisions around care at the end of life. Patients who lack capacity may require proxy decision-makers, whether familial or legal, to be involved in care decisions [24]. Xie [25], in exploring end-of-life decision-making models in people living with dementia, highlighted a lack of current tools that allow values and preferences to be incorporated in decision-making and a lack of sensitivity to cultural variance. A one-size-fits-all approach to end-of-life planning and decision-making is not effective for patients or caregivers [20], and individuality and diversity must be taken into account to serve patients and their families’ best. COVID-19 has resulted in disproportionately higher mortality in minority ethnic communities. Thus, culturally tailored decision-making interventions and approaches are particularly important in order to enable diverse patients and caregivers to plan and make good care and end-of-life decisions.

### Advance care planning

ACP is a generally welcomed process in nursing homes and the community setting [26]. There is, however, limited evidence that advance care plans support proxies to make consistent healthcare decisions, and evidence showing good accord between patient and proxy decisions is lacking [27]. There is also evidence that ACP engagement and comprehension levels may differ significantly according to contextual factors such as income level [28], patient attitudes, comfort and level of trust in the healthcare system [29]. In the UK, capacity legislation is a mandatory part of the decision-making process that must be included. Though ACP is seen as aspirational, care must be taken to ensure that access to ACP services and interventions is culturally appropriate. ACP should be an iterative process, based on patient values rather than set treatment options, integrated across the illness trajectory and continually reviewed [30,31]. During the COVID-19 pandemic, advance care plans may need to be renegotiated and reconsidered as care options change—for example, a person who wants to die in hospice may reconsider this decision in light of visitors not being allowed. Thus, the continual review element of ACP is particularly important within the COVID-19 context.

### Decision-making model

**Figure 2:**
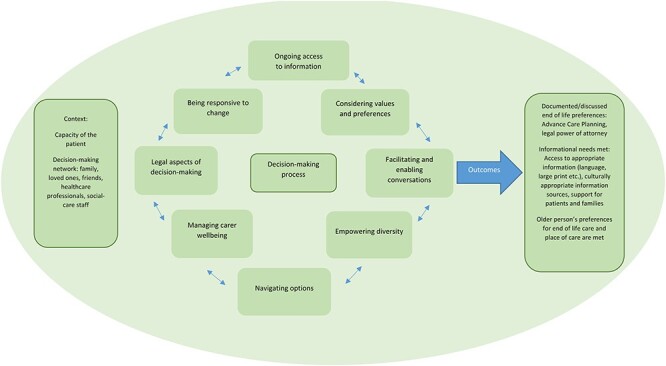
Decision-making model.

Through reviewing the existing literature, we found that certain values and processes were common throughout the process of decision-making [15–20]. In light of this, a decision-making model was devised (Figure 2). The model highlights different needs and aspects of decision-making, from considering informational, access and cultural needs to facilitating conversations and access to legally appropriate formal options such as ACP and power of attorney. Considering these separate aspects explicitly may help better define concrete outcomes such as PoC and death, legal aspects of dealing with diminished capacity and better providing for diverse informational and support needs. The process as a whole, including outcomes, should be iterative and reviewed regularly to ensure ongoing appropriateness.

The concept of shared decision-making was prevalent, with a range of respondents (general public, carers and older people) talking about making care decisions alongside their families, friends and care providers. Thus, this formed the overarching context of the decision-making model—with a person’s social, support and (chosen) family group being the baseline that decision-making starts from, together with the capacity of the individual. The aspects that form a circle are iterative and necessary parts of the decision-making process. Not all aspects will be applicable to all situations and circumstances, though each should be approached and assessed deliberately to determine this. These factors will then work in conjunction to shape decision-making, from identifying available options to putting legal and practical frameworks in place to facilitate older adults to be cared for and die as they wish—as seen in the box on the right.

During the COVID-19 pandemic, this decision-making model can operate in the same way as it would outwith the pandemic. As external circumstances change, the range of information, contexts and options that can be considered by the model also change—and the process remains the same.

## Conclusions

The process and outcomes of decision-making for older people are affected by many factors—all of which have the potential to influence both patients and caregivers experience of illness and dying. Within the context of COVID-19, such decisions may have to be made rapidly and be reflexive to changing needs. These include needs of systems themselves, such as modified triage and service allocation, as well as individual and family desires. ACP and decision-making aids can help to facilitate patients and caregivers to make choices around issues such as modified visiting rules, availability and appropriateness of domiciliary care, multigenerational caring needs and rapid changes of circumstance. The combination of the current pandemic context and lessons-learned from non-pandemic care planning has a number of implications for future best-practice.

## Appendix 1: Search Strategy

Search date: 22nd April 2020.

((Adult or Adults) and (End of Life Care or Terminal Care or Palliative Care or palliat^*^ or hospice) and (pref^*^ or wish^*^ or choice^*^ or chose^*^ or decision^*^ or decid^*^) and (place^*^ or location^*^ or setting or where) and (Death or dies or die or dying or died) and Review).

(‘Adult’[MeSH Terms] OR ‘Adult’[All Fields] OR ‘Adults’[All Fields]) AND (‘End of Life Care’[All Fields] OR ‘Terminal Care’[MeSH Terms] OR ‘Terminal Care’[All Fields] OR ‘Palliative Care’[MeSH Terms] OR ‘palliat^*^’[All Fields] OR ‘terminal^*^’[All Fields] OR ‘endstage’[All Fields] OR ‘Death’[MeSH Terms] OR ‘hospice^*^’[All Fields] OR ‘Death’[All Fields]) AND (‘pref^*^’[All Fields] OR ‘wish^*^’[All Fields] OR ‘choice^*^’[All Fields] OR ‘chose^*^’[All Fields] OR ‘decision^*^’[All Fields] OR ‘decid^*^’[All Fields]) AND (‘place^*^’[All Fields] OR ‘location^*^’[All Fields] OR ‘setting’[All Fields] OR ‘where’[All Fields]) AND (‘death’[All Fields] OR ‘Death’[MeSH Terms] OR ‘dies’[All Fields] OR ‘die’[All Fields] OR ‘dying’[All Fields] OR ‘died’[All Fields]) AND Review[ptyp].
